# SUCCESSFULLY “AGING ALONE”? UNEQUAL OPPORTUNITIES AND RISING RISKS IN FAMILY-BASED MODELS OF CARE CROSS-NATIONALLY

**DOI:** 10.1093/geroni/igae098.1892

**Published:** 2024-12-31

**Authors:** Christine Mair

**Affiliations:** University of Maryland, Baltimore County, Baltimore, Maryland, United States

## Abstract

For the first time in human history, older adults will outnumber children and a substantial and growing proportion will live alone and lack one or more nuclear family tie. Such unprecedented shifts require a reevaluation of existing models of “successful aging”, particularly in terms of long-term care policies. This paper draws on country-level data from multiple publicly available sources (e.g., World Bank, Organization for Economic Cooperation and Development, Our World in Data, and the World Values Survey) to examine cross-national patterns of development, health, demography, resources and policies, and cultural values in low-, middle-, and high-income countries. Although there exists substantial heterogeneity across countries, country-level patterns illustrate the economic privilege of living alone and the dominance of “successful aging” opportunities in high income countries. Cultural values about family reflect standard patterns of economic development, yet friendship emerges as a particularly consistent global value. Living alone is associated with poor health in lower income countries, yet better health in higher income countries. Aging “alone” is a risk factor in some contexts, yet a marker of privilege in others. Models of “successful aging” are largely unobtainable in lower income countries, and therefore require a thorough incorporation of global realities, or final abandonment in favor of more nuanced structural perspectives. Long-term care policies that assume the presence of family will yield increasing risk over time across all global contexts and represent a key vulnerability in the future of healthy aging policy.

